# Predictors of Neurodevelopment in Microcephaly Associated with Congenital Zika Syndrome: A Prospective Study

**DOI:** 10.3390/children10121831

**Published:** 2023-11-21

**Authors:** Adriana M. Mattos, Valmir N. Rastely-Junior, Matheus M. Pires, Juan P. Aguilar, Millani S. A. Lessa, Clarina Regis, Mariana Wanderley, Julio Leony, Joseane Bouzon, Verena Ballalai, Carina Vieira, Gustavo B. S. Carvalho, João R. M. Almeida, Nivison Nery, Rodrigo Leal, Federico Costa, Albert I. Ko, Mitermayer G. Reis, Jamary Oliveira-Filho

**Affiliations:** 1 Hospital Geral Roberto Santos, Salvador 40301-110, Brazil; valmir.j.rastely@gmail.com (V.N.R.-J.); regis.clarinadoc@gmail.com (C.R.); marianaaraujo15.1@bahiana.edu.br (M.W.); julioleony15.1@bahiana.edu.br (J.L.); vballalai@gmail.com (V.B.); carinamvieira@yahoo.com.br (C.V.); gustavobalthazar@gmail.com (G.B.S.C.); 2 Post-Graduate Program in Health Sciences, Federal University of Bahia, Salvador 40170-110, Brazil; matheusmpires@yahoo.com.br (M.M.P.); fonobouzon@bol.com.br (J.B.); peres@mail.com (R.L.); jamary@mail.harvard.edu (J.O.-F.); 3 Post-Graduate Program in Public Health, Institute of Collective Health (ISC), Federal University of Bahia, Salvador 40170-110, Brazil; pkjpablo@gmail.com (J.P.A.); mila.misoual@gmail.com (M.S.A.L.); nivisonjr@hotmail.com (N.N.J.); fcosta2001@gmail.com (F.C.); 4 Hospital Professor Edgard Santos, Federal University of Bahia, Salvador 40170-110, Brazil; jrmaltez.a@gmail.com; 5 Gonçalo Moniz Institute, Foundation Oswaldo Cruz, Salvador 40296-710, Brazil; mitermayer.reis@fiocruz.br; 6 Department of Epidemiology of Microbial Diseases, Yale School of Public Health, New Haven, CT 06510, USA; albert.ko@yale.edu

**Keywords:** Zika virus, microcephaly, child development, developmental disabilities

## Abstract

The municipality of Salvador, situated in Brazil, distinguished itself as the epicenter of the emergence of microcephaly related to congenital manifestations of Zika syndrome. Despite the anticipated significant developmental setbacks in these children, research has indicated a varied range of outcomes, with certain instances even reflecting minimal developmental delay. Our objective was to pinpoint determinants that could forecast developmental anomalies in children diagnosed with microcephaly associated with congenital Zika syndrome (CZS). Methodology: A forward-looking clinical and neurodevelopmental examination was conducted focusing on neonates diagnosed with microcephaly with CZS, birthed between September 2015 and April 2016 at the Hospital Geral Roberto Santos, in Salvador city. That infants were monitored up to their third year by a multiprofessional team. Child development was assessed using the composite Bayley III score. Undertaken by two blinded experts, cranial CT scan analysis was performed during the neonate period for the detection of brain abnormalities and to quantify ventricle enlargement, measured by Evans’ index (EI). Results: Fifty newborns were evaluated with a median head circumference of 28 cm (interquartile range 27–31 cm). EI was associated with neurodevelopmental delay at three years and remained significant after adjustment for head circumference. A 0.1-point increase in EI was associated with a delay of 3.2 months in the receptive language (*p* = 0.016), 3.4 months in the expressive language (*p* = 0.016), 3.4 months in the cognitive (*p* = 0.016), 2.37 months in the gross motor (*p* = 0.026), and 3.1 months in the fine motor (*p* = 0.021) domains. Conclusions: EI predicted neurodevelopmental delay in all Bayley domains in children with microcephaly associated with CZS.

## 1. Introduction

Zika virus (ZIKV) infection is an emergent infection, described for the first time in Africa as an arboviral febrile disease with rash. Since 2015, it has gained prominence due to its association with serious neurological abnormalities among the infants born to pregnant women in the Americas during the epidemic spread of the disease [1,2]. The state of Bahia was the epicenter of ZIKV in Brazil, with symptoms similar to other arboviruses [3]. Following the initial outbreak, an increasing number of newborns were diagnosed with microcephaly and other neurological malformations associated with ZIKV infection, currently known as congenital Zika syndrome (CZS) [3,4,5,6].

The spectrum of findings in CZS ranges from microcephaly, defects in brain development, arthrogryposis, and ophthalmic abnormalities secondary to the effect of intrauterine Zika virus infection [2,3,4,5,6,7]. Microcephaly results from cerebral malformation and is identified when the head circumference (HC) of newborns is less than 2 standard deviations (SDs) based on the World Health Organization (WHO) child growth standards (WHO table) or the Intergrowth-21st, in preterm infants, with measurements adjusted to gestational age and sex [8].

Despite the fact that congenital exposure and microcephaly in children is associated with neurodevelopmental delay, some studies have described a heterogeneity in clinical presentations [9,10,11,12,13,14,15], including neurological sequelae such as neurosensory disorders, dysphagia, and epilepsy [16]. Although some studies have identified qualitative clinical and radiological prognostic factors for neurological outcomes in children with microcephaly [17,18], little is known about quantitative predictive factors for long-term development in cases associated with CZS.

In this study, we aimed to identify clinical and radiological markers that would serve as quantitative and qualitative predictive factors for long-term developmental disorders in children with microcephaly associated with CZS. Therefore, what are the clinical and neuroimaging findings associated with developmental delay in children with microcephaly associated with CZS?

## 2. Methods

### 2.1. Patients and Definitions

We conducted a prospective study following all newborns with microcephaly associated with CZS, born at Hospital Geral Roberto Santos, a tertiary hospital in the city of Salvador, in the state of Bahia, Brazil, during the outbreak of ZIKV infection between September of 2015 and April of 2016.

Microcephaly was defined as a head circumference in centimeter (HC) at birth < −2.00 SD (standard deviation) for age and sex based on the World Health Organization (WHO) child growth standard (WHO table) or the Intergrowth-21st standard if infants were born preterm. Severe microcephaly was defined as HC < −3.00 SD for age and sex, using the same growth standards [8].

CZS associated with microcephaly included microcephaly with laboratory-confirmed cases of CZS and highly probable CZS when there were microcephaly and at least two of the following findings: arthrogryposis (congenital contractures), head computed tomography (CT) with characteristic brain abnormalities (cerebral intraparenchymatous calcifications, especially subcortical calcifications, corpus callosum abnormalities, ventriculomegaly, or neuronal migration disorders such as lissencephaly, pachygyria, polymicrogyria, or heterotopia), or ophthalmic abnormalities (pigmented retinal mottling, chorioretinal atrophy, macular scarring, glaucoma, optic nerve atrophy and abnormalities, intraocular calcifications, microphthalmia, anophthalmia, iris coloboma, lens subluxation) [2,19]. Children with evidence of genetic syndrome, other congenital infections (by serological tests for toxoplasmosis, cytomegalovirus, syphilis, or rubella), or any central nervous system malformation not associated with CZS were excluded. Serological tests were performed using enzyme-linked immunosorbent assay (ELISA) for detecting anti-ZIKV IgM and IgG antibodies (IgM-ELISA umbilical cord and peripheral blood testing at birth and/or blockage of binding (BOB) IgG ELISA for mothers and children at >6 months of age). When available, molecular tests by reverse transcription polymerase chain reaction (RT-PCR) and the plaque reduction neutralization test (PRNT90) were also performed for the laboratorial confirmation of congenital ZIKV exposure. The study was approved by the hospital’s Ethics in Clinical Research committee under the number 1,866,918. Written informed consent was obtained from the parents of all studied patients.

### 2.2. Clinical Evaluation

All children included in this prospective study were followed at least every 3 to 6 months in a multiprofessional outpatient clinic which included pediatric and neuropediatric evaluation, motor physiotherapy, occupational therapy, and speech therapy. In each visit, infants underwent a full neurologic examination and standardized growth parameter measurements (head circumference, weight, and length). A systematic neurodevelopmental evaluation at each routine visit was performed to investigate clinical progress and identify any delay or other abnormalities, such as sleep problems, irritability, seizures, or evidence of feeding or swallowing dysfunction.

The maternal history included the mother’s age, the month of infection, and information related to prenatal care. The report of Zika infection include rash and fever symptoms. Newborn data included gestational age, perinatal complications, weight, length, and HC at birth.

Head CT scan was performed during the neonatal period, before hospital discharge, and evaluated by two investigators who were blinded to patients’ clinical data. Findings were classified according to the presence of cerebral intraparenchymatous calcifications, dysgenesis of the corpus callosum, and neuronal migration disorders. Divergent ratings were resolved by consensus. The severity of ventriculomegaly was quantified by the measurement of Evans’ index (EI). It is a linear index for ventricular measurement, calculated by the ratio of the frontal horn diameter to the largest internal cranial diameter. Inter-rater agreement was calculated for each categorical (kappa statistics) and continuous (intraclass correlation coefficient) variable.

Neurodevelopment was assessed by the Bayley Scales of Infant and Toddler Development (Bayley–III), between 36 and 42 months of age, comparing raw and compositive scores to assess three domains (cognitive, language, and motor functions).

The Bayley Scales are classified as extremely low by composite scores less than 69; borderline between 70 and 79; low average between 80 and 89; average between 90 and 109; high average between 110 and 119; superior between 120 and 129; very superior between 130 and above. Since most of the cases analyzed in our study presented with severe delay, we chose to evaluate neurodevelopment as a linear outcome rather than a dichotomous one. In order to increase clinical applicability, we transformed the raw Bayley-III scores into developmental delay measured in months (calculated as the delay in months between chronological age at the time of evaluation and the developmental age equivalents for the tasks performed in the Bayley-III score system).

Due to the inherent nature of an epidemic and its time-sensitive pattern of evolution throughout the community, the study used a convenience sample consisting of all patients with suspicion of microcephaly referred to the hospital that fulfilled the inclusion criteria. The multiprofessional outpatient clinic was the primary reference center of evaluation for the microcephaly associated with CZS in the state of Bahia and evaluated most of the cases that occurred during the outbreak.

### 2.3. Analysis

Categorical variables were expressed as numbers and percentages. Continuous variables were expressed as means and SDs if normally distributed (assessed by the Shapiro–Wilk test), or as medians and interquartile ranges (IQRs) if non-normally distributed. The linear correlation between two continuous variables was evaluated using the Pearson (r) or Spearman (rs) correlation coefficient, as appropriate. Linear regression was used for univariate analyses between each independent variable and developmental delay in months (dependent variable). Any significant (*p* < 0.05) variable in univariate analyses was included in a multivariate regression model adjusted for the HC Intergrowth Z-score. All statistical analyses were performed using SPSS version 20 (IBM, Armonk, New York, NY, USA), and a *p*-value < 0.05 was considered to indicate significance in the final linear regression model.

## 3. Results

**Figure 1:**
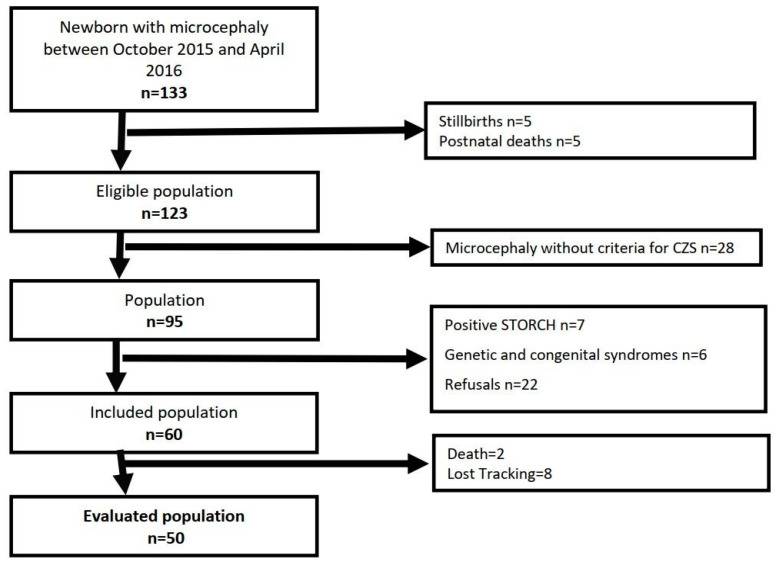
Flow of patients through the study.

From 30 September 2015 to 30 April 2016, a total of 133/1699 (7.8%) children were born with microcephaly at Hospital Geral Roberto Santos in Salvador, Bahia state, in the northeast region of Brazil. After formal evaluation, 60/133 (45.1%) fulfilled predefined criteria for CZS, but 2 children died and 8 were lost to follow-up (Figure 1). The remaining 50 children had mothers with a mean age of 26 ± 6 years old that reported full prenatal care (84%) and Zika infection symptoms during pregnancy (86%). Demographic and children’s clinical characteristics at baseline are presented in Table 1. The main measurements at birth in the studied population were mean weight, 2492 g (+/−547); median length, 46.0 cm (IQR = 43.8–48.3); and median HC, 28 cm (IQR = 27−31), with a mean Intergrowth Z-score of −3.7 (±1.5). Arthrogryposis was present in seven patients (14%), and some complications in the neonatal period included seizure (22%), dysphagia (14%), jaundice (10%), and respiratory distress (2%).

The median age on radiological examination was 13 (IQR = 3–68) days, and all patients presented classical characteristics of CZS on head CT (Table 1). The most prevalent findings were ventriculomegaly (78%), cerebral intraparenchymatous calcifications (80%), and neuronal migration disorders (68%). The median value for Evans´ index was 0.39 (IQR = 0.35–0.44).

Neurodevelopmental assessment, between 36 and 42 months, showed extremely poor performance in the cognitive domains in 86%, in language domains in 84%, and in motor domains in 88% of the studied patients. The results of the Bayley-III according to the equivalent age are shown in Table 2. The mean developmental delays were 26.8 months for the cognitive, 25.7 for the receptive language, 27.1 for the expressive language, 26.8 for the fine motor, and 28.9 for the gross motor domains. 

**Figure 2:**
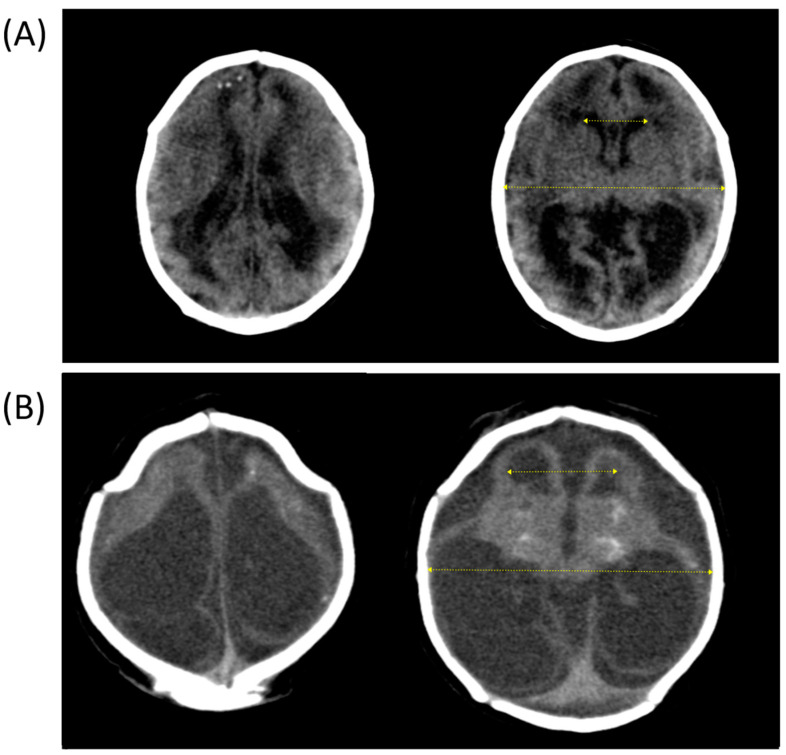
Head computed tomography showing the difference in the degree of brain damage between two children and their respective delays on Bayley Scales. (**A**) Evans’ index 0.32; delay of 23.9 months in cognitive, 22.9 months in receptive language, 23.0 months in expressive language, 22.0 months in fine motor, and 21.0 months in gross motor domains. (**B**) Evans’ index 0.41; delay of 41.8 months in cognitive, 39.9 months in receptive language, 41.8 months in expressive language, 41.8 months in fine motor, and 41.8 months in gross motor domains.

The EI (Figure 2) was the only variable associated with developmental delay and remained significant after adjustment for HC Intergrowth Z-score. A 0.1-point increase in Evans’ index was associated with a mean delay of 3.4 months in the cognitive, 3.2 months in the receptive language, 3.4 months in the expressive language, 3.1 months in the fine motor, and 2.4 months in the gross motor domains (Table 3). Complete linear regression models for each domain are shown in the Supplementary Tables.

## 4. Discussion

The purpose of this report is to provide information that can help pediatricians to identify a greater risk of development delay, which may help to decide the best clinical management strategy in children with CZS-associated microcephaly and improve prognostication based on clinical and neuroimaging neonatal findings. The benefits of a precise prediction of risk for abnormal neurodevelopment include the opportunity to initiate individualized and targeted rehabilitation. In addition, as discussed in a literature review, examining the spectrum of CZS can help identify markers to provide better family counseling [18].

Our results demonstrate that the degree of ventricular dilatation measured by EI on CT brains in the neonatal period can predict worst long-term neurodevelopmental outcomes in patients who have microcephaly associated with CZS. Congenital Zika syndrome is associated with severe brain damage resulting in poor neurodevelopment, motor and hearing impairment, epilepsy, swallowing difficulties, and low vision [2,11,19,20,21]. The symptoms are likely correlated to the severity of brain abnormalities [16,20], similarly to other symptomatic TORCHS infections [20]. In one report, lower than expected HC in children with cerebral palsy and probable CZS was associated with impaired neurodevelopment on Bayley-III and poor prognosis for independent walking [9]. A long-term cohort that included 24 children, between 18 and 24 months of age, demonstrated profound developmental delays across all functional domains with a mean equivalent developmental age between 2 and 4 months [22].

When compared to other causes of microcephaly, CZS seems to show more severe neurodevelopmental delays. In infants with microcephaly from different etiologies, a retrospective review identified longitudinal neurodevelopmental delay outcomes [23]. By 27 months of age, 73% of the children had a delay in one or more areas of development which included the gross motor domain (65%), visual–motor domain (59%), and language domain (59%). The authors concluded that infants with microcephaly are at significant risk for delay across all aspects of development and for long-term disability [23]. In our study, over 80% of the infants had some form of developmental delay in every analyzed domain. An interesting prospective cohort study reported that out of the 216 infants exposed to ZIKV during pregnancy, 31% had abnormal neurodevelopmental scores on the Bayley test [15]. However, the limitations of the study may have led to inaccuracy in measuring the contribution of the effects of ZIKV infection on the observed outcomes. Firstly, asymptomatic children (without clinical and radiological evidence of ZIKV infection) whose mothers had positive laboratory tests for ZIKV during pregnancy were considered exposed to ZIKV, but without presenting or considering the results of children laboratory tests for ZIKV. In the cases of maternal ZIKV infection during pregnancy, only 26% of fetuses with congenital exposure will be infected with ZIKV through vertical transmission [24]. Secondly, the cohort study did not consider the exclusion or effect of other confounding factors, such as other maternal coinfections.

Other congenital infection impairments are usually less severe than CZS. The most similar in severity disability can be seen in congenital cytomegalovirus (CMV) infection. The long-term outcomes of symptomatic congenital CMV infection show microcephaly in 70%, mental retardation in 61%, hearing loss in 30%, neuromuscular disorders in 35%, and chorioretinitis or optic atrophy in 22% of patients, with a high risk for significantly impaired development [25]. Congenital CMV magnetic resonance imaging (MRI) findings are also similar, showing ventriculomegaly, polymicrogyria, and white matter abnormalities that correlate significantly with poor neurological prognosis. CT findings, such as cerebral intraparenchymatous calcifications, ventriculomegaly, and brain atrophy, have high specificity as predictors of neurological impairment and developmental delay [25,26,27].

Neuroimaging findings in CZS have been extensively described, but most lack specificity to predict abnormal neurodevelopment. Furthermore, Zika virus can cause congenital brain damage even in newborns without microcephaly [28], suggesting that the full spectrum of this disorder is still unknown. A retrospective cohort study of 110 children with CZS who exhibited neuroimaging abnormalities at birth, such as calcifications and cortical malformations, observed that they were more likely to exhibit clinical findings, which included congenital contracture and ophthalmologic and hearing abnormalities, than children without these neuroimaging findings [17]. These findings, along with the results of our study, reemphasize the importance of imaging studies as part of the initial evaluation and for the follow-up of children with congenital exposure to ZIKV.

Overall, the description and frequency of brain abnormalities in previous cohorts are very similar to our study. They mostly show brain atrophy, ventriculomegaly, malformations of cortical development, cortical and subcortical calcifications, craniofacial disproportion, microcephaly, agenesis/hypoplasia of the corpus callosum, cerebellar and brainstem hypoplasia, and schizencephaly [29,30,31,32].

In our study, we found that the severity of ventricular enlargement was the most important predictor of impaired neurodevelopment. The Evans’ index is the ratio of the transverse diameter of the anterior horns of the lateral ventricles to the greatest internal diameter of the skull. It was initially described in 1942 for estimating the severity of ventricular enlargement, cerebral atrophy, and hydrocephalus via encephalography [33]. In 1976, it was transposed to computed tomography [34] and has been extensively used as an indirect marker of ventricular volume for hydrocephalus clinical management and other neurological and neurosurgical disorders [35,36]. Although more precise techniques are currently described, such as ventricle volume measurement on high-resolution T1 MRI scans or head CT [36], they are not usually widely available.

Linear indices for ventricular measurement, such as the EI, the frontal horn index, and the bicaudate index, can be straightforwardly estimated on 2D ultrasound, CT, or MRI and may, therefore, be more practical to use as a prognostic tool. Linear indices are easy-to-use, accessible, cost-effective, and reliable as surrogates of ventricular size in pediatric clinical practice [37,38]. Evans’ index, in particular, might provide a conservative estimate of ventriculomegaly, since it does not account for the asymmetrical dilation of other ventricular horns. Nevertheless, we chose to apply it due to its convenience and reproducibility in different imaging methods.

International guidelines define ventricular enlargement as an EI larger than 0.3, decreasing minimally with age [39]. In children, the mean EI reference value was 0.263 ± 0.034 in a pediatric population from Silesia [38]. Another study found an EI of 0.263 (±0.005) in females and 0.282 (±0.008) in males in the zero to twelve months group [36]. They also found that changes between consecutive age groups were not statistically significant [38].

In children, changes in the ventricular system are a consequence of high hydrostatic pressure (hydrocephalus) or of brain atrophy related to many pathological factors that can influence the child development [38]. In our study, all cases were suggestive of ventricular dilatation secondary to brain atrophy. Therefore, EI could be used as a surrogate marker for global cerebral damage secondary to congenital ZIKV infection.

We did not evaluate serial neuroimaging results; therefore, it is possible that the EI might have increased over the evaluated period in a proportion of cases. There is some evidence that ZIKV continues to replicate in fetal brains during the first months of extra-uterine life [40,41]. Similarly to other congenital infection syndromes, some patients with microcephaly associated with CZS develop hydrocephalus, but there are no data evaluating the increase in non-hypertensive ventriculomegaly, which could correspond to a progression of the virus aggression and worst prognosis.

In CZS and other infectious congenital syndromes, categorical variables have been mostly used as predictors of neurodevelopment, but quantitative variables have seldom been used to correlate with the severity of neurological impairment. This study has some limitations. Our study did not include newborns with normal head sizes. It would be interesting to investigate which neuroimaging findings could predict neurodevelopmental outcomes in these patients as well. The absence of a healthy control group in our study is primarily due to the ethical concerns associated with conducting neuroimaging studies, such as computed tomography, on healthy children. Furthermore, obtaining ethical approval and parental consent for such procedures on healthy minors can be particularly challenging. Due to this study design, we also did not collect data on neurological scales in a normal control population, although each clinical scale does have studies on normal values for this particular age group. Our research aims to delve into the neurologic and neuroanatomic predictors within the specific population of children affected by microcephaly due to Zika exposure, making the internal comparison within this group crucial to understanding the heterogeneous neurodevelopment observed.

In conclusion, in children with microcephaly associated with CZS, a simple quantitative neonatal radiological finding obtained in the neonatal period (Evans’ index), which reflects the degree of ventricular dilatation, is associated, and can predict more severe neurodevelopmental impairments for this population by the age of three years. This radiological finding can be obtained with other widely available imaging techniques, such as ultrasound scans, and may be suited as an early prognostic predictor to provide individualized care and rehabilitation.

## Figures and Tables

**Table 1:** Demographic, clinical, and radiological characteristics of infants with microcephaly associated with congenital Zika syndrome at baseline.

Variable	Values, *n* (%) or Mean +/− Standard Deviation or Median [Interquartile Range]
**Maternal characteristics**	
Age (years) *	26 ± 6
Symptomatic Zika infection during pregnancy	43 (86)
Symptoms during first trimester	33 (76.7)
Complete prenatal visits	42 (84)
**Newborn clinical characteristics**	
Gestational age ^‡^	39 [37–40]
Apgar 1’ ≥ 8	50 (100)
Apgar: 5’ ≥ 8	50 (100)
Birth weight (g) *	2492 ± 547
Birth length (cm) ^‡^	46 [43.8–48.3]
Birth HC (cm) ^‡^	28 [27–31]
Intergrowth-21st (Z score) *	−3.7 ± 1.5
Female sex	27 (54)
Arthrogryposis	7 (14)
Low vision Neonatal seizure	46 (92) 11 (22)
Neonatal jaundice	5 (10)
Respiratory distress	1 (2)
Neonatal dysphagia	7 (14)
**Radiological characteristics**	
CT scan—infant age (in days) ^‡^	13 [3–68]
Evans’ index ^‡^	0.39 [0.35–0.44]
Ventriculomegaly	39 (78)
Cerebral intraparenchymatous calcifications	40 (80)
Neuronal migration disorders	34 (68)
Cerebellum hypoplasia	4 (8)
Corpus callosum anomaly	20 (40)
**ZIKV laboratory results**	
Positive anti-ZIKV IgM (n/N)	24/40 (60%)
Positive anti-ZIKV IgG (n/N)	42/50 (84%)
Positive ZIKV RT-PCR (n/N)	16/37 (43%)
Positive ZIKV PRNT90 (n/N)	33/33 (100%)

* expressed as mean and standard deviation, ^‡^ expressed as median and interquartile. HC: head circumference.

**Table 2:** Neurological and neurodevelopmental evaluation in infants with microcephaly associated with congenital Zika syndrome according to the Bayley Scales.

Cognitive Score	Extremely Low 42 (85.7) Low Average 1 (2)
Language score	Borderline 1 (2) Extremely low 41 (83.7) Low average 1 (2)
Motor score	Extremely low 42 (85.7) Low average 1 (2)
Delay in months—Cognitive domain	26.8 [35.8–22.9]
Receptive language domain	25.7 [32.1–20.9]
Expressive language domain	27.1 [34.3–22.6]
Fine motor function domain	26.8 [36.1–23.9]
Gross motor function domain	28.9 [36.4–22.9]

Bayley Scales composite scores: 69 and below = extremely low; 70–79 = borderline; 80–89 = low average; 90–109 = average; 110–119 = high average; 120–129 = superior; 130 and above = very superior.

**Table 3:** Variables associated with developmental delay in months in Bayley Scales of infants with microcephaly associated with congenital Zika syndrome.

Domain	Variables	Adjusted Effect (Delay in Months)	IC 95%	*p*
Cognitive	zHC at birth Evans’ index *	1.48 3.42	–0.70–3.70 6.6–61.8	0.186 0.016
Receptive language	zHC at birth Evans’ index *	1.70 3.21	–0.38–3.78 6.4–57.9	0.106 0.016
Expressive language	zHC at birth Evans’ index *	1.95 3.42	–0.20–4.12 6.6–61.8	0.074 0.016
Gross motor	zHC at birth Evans’ index *	1.44 2.37	–0.23–3.12 3.0–44.4	0.088 0.026
Fine motor	zHC at birth Evans’ index *	1.48 3.14	–0.65–3.61 5.0–57.8	0.169 0.021

* per 0.1 increase in Evans’ index. zHC: Head Circumference Intergrowth Z-score.

## Data Availability

Data are contained within the article and Supplementary Materials.

## Supplementary Materials

The following supporting information can be downloaded at: https://www.mdpi.com/article/10.3390/children10121831/s1. Table S1. Variables associated with developmental delay in cognitive domain in infants with microcephaly. Table S2. Variable associated with developmental delay in receptive language domain in infants with microcephaly related to congenital Zika syndrome. Table S3. Variables associated with developmental delay in expressive language domain in infants with microcephaly related to congenital Zika syndrome. Table S4. Variables associated with developmental delay in gross motor domain in infants with microcephaly related to congenital Zika syndrome. Table S5. Variables associated with developmental delay in fine motor in infants with microcephaly related to congenital Zika syndrome.
