# Supplementary material for: Predictors of Neurodevelopment in Microcephaly Associated with Congenital Zika Syndrome: A Prospective Study
Source: Children (Basel). 2023 Nov 21;10(12):1831. doi: 10.3390/children10121831 (PMC10741834; doi:10.3390/children10121831)
Supplement: Supplementary file 1 [file children-10-01831-s001.zip › children-2662076-supplementary.pdf]

Supplementary tables:

Table S1. Variables associated with developmental delay in cognitive domain in infants with microcephaly

Variables	Unadjusted effects	IC 95%	p	Adjusted effects	IC 95%	p
Mother age (years)	0.168	(-0.36 – 0.70)	0.53			
Maternal symptoms	-7.03	(-16.35 – 2.29)	0.14			
Gestacional age (weeks)	-0.80	(-2.14 – 0.55)	0.24			
Birth weight (g)	0.0	(-0.006 – 0.007)	0.91			
Length at birth (cm)	-0.23	(-1.18 – 0.72)	0.62			
HC at birth (cm)	-0.24	(-1.7 – 1.22)	0.74			
zHC at birth	0.94	(-1.24 – 3.11)	0.39	1.48	(-0.7 – 3.7)	0.186
Apgar 1'	0.27	(-2.01 – 2.56)	0.81			
Apgar 5'	-4.15	(-10.87– 2.58)	0.22			
Female Sex	2.64	(-3.77 –9.05)	0.41			
Arthrogryposis	1.02	(-8.54 – 10.57)	0.83			
Low vision	0.45	(-6.15 – 7.04)	0.89			
Neonatal seizure	5.87	(-1.72 – 13.47)	0.13			
Neonatal jaundice	4.32	(-5.94 – 14.58)	0.40			
Neonatal dysphagia	0.67	(-8.89 – 9.03)	0.99			
Ventriculomegaly	-1.21	(-9.32 – 6.89)	0.76			
Colpocephaly	5.24	(6.07 – 16.56)	0.36			
Corpus callosum dysgenesis	3.32	(-3.14 – 9.78)	0.31			
Cerebellar hypoplasia	7.47	(-3.72 – 18.7)	0.19			
Evans' Index*	2.802	(0.1156 – 5.443)	**0.038**	**3.42**	**(6.6 – 61.8)**	**0.016**

***per 0.1 increase in Evans' Index.**

Table S2. Variable associated with developmental delay in receptive language domain in infants with microcephaly related to congenital Zika syndrome.

**Variables**	**Unadjusted effects**	**IC 95%**	**p**	**Adjusted effects**	**IC 95%**	**p**
Mother age (years)	0.19	(-0.31 – 0.68)	0.46			
Maternal symptoms	-6.34	(-15.09 – 2.41)	0.15			
Gestacional age (weeks)	-0.86	(-2.109 – 0.394)	0.174			
Birth Weight (g)	0.001	(-0.005 – 0.007)	0.820			
Length at birth (cm)	0.14	(-0.75 – 1.03)	0.751			
HC at birth (cm)	-0.98	(-1.47 – 1.28)	0.886			
zHC at birth	1.18	(-0.85 – 3.20)	0.248	1.701	(-0.38 – 3.78)	0.106
Apgar 1'	0.52	(-1.61 – 2.66)	0.624			
Apgar 5'	-4.49	(-10.75 – 1.77)	0.155			
Female Sex	2.52	(-3.49 – 8.53)	0.402			
Arthrogryposis	0.22	(-8.74 – 9.18)	0.960			
Low vision	0.58	(-6.76 – 5.60)	0.851			
Neonatal seizure	4.49	(-2.70 – 11.67)	0.215			
Neonatal jaundice	4.94	(-4.64 – 14.52)	0.305			
Neonatal dysphagia	0.15	(-8.55 – 8.25)	0.972			
Ventriculomegaly	1.49	(-9.09 – 6.09)	0.693			
Colpocephaly	3.545	(-7.112 – 14.201)	0.506			
Corpus callosum dysgenesis	2.73	(-3.34 – 8.905)	0.37			
Cerebellar hypoplasia	5.954	(-4.60 – -16.51)	0.262			
Evans' Index*	2.5264	(0.029 – 5.023)	**0.047**	**3.2142**	**(6.38 – 57.9)**	**0.016**

***per 0.1 increase in Evans' Index.**

Table S3. Variables associated with developmental delay in expressive language domain in infants with microcephaly related to congenital Zika syndrome.

**Variables**	**Unadjusted effects**	**IC 95%**	**p**	**Adjusted effects**	**IC 95%**	**p**
Mother age (years)	0.12	(-0.41 – 0.642)	0.66			
Maternal symptoms	-7.96	(-17.053 – 1.112)	0.09			
Gestacional age (weeks)	-0.88	(-2.20 – 0.423)	0.18			
Birth weight (g)	0.001	(-0.005 – 0.007)	0.76			
Length at birth (cm)	-0.18	(-1.11 – 0.750)	0.69			
HC at birth (cm)	0.48	(-1.39 – 1.49)	0.95			
zHC at birth	1.44	(-0.676 – 3.555)	0.18	1.95	(-0.203 – 4.12)	0.074
Apgar 1'	0.10	(-2.15 – 2.35)	0.93			
Apgar 5'	-4.30	(-10.91 – 2.31)	0.196			
Female Sex	2.12	(-4.2 – 8.44)	0.50			
Arthrogryposis	0.23	(-9.105 – 9.701)	0.95			
Low vision	0.89	(-5.597 –7.370)	0.784			
Neonatal seizure	4.25	(-3.31 – 11.83)	0.26			
Neonatal jaundice	4.95	(-5.11 – 15.01)	0.33			
Neonatal dysphagia	0.38	(-8.43 – 9.20)	0.93			
Ventriculomegaly	-2.13	(-10.09 – 5.82)	0.53			
Colpocephaly	3.93	(-7.24 – 15.11)	0.48			
Corpus callosum dysgenesis	2.90	(-3.46 – 9.27)	0.36			
Cerebellar hypoplasia	6.56	(-4.498 – 17.62)	0.238			
Evans' Index*	2.29	(-0.13 – 4.89)	0.083	**3.42**	**(6.6 – 61.8)**	**0.016**

***per 0.1 increase in Evans' Index.**

Table S4. Variables associated with developmental delay in gross motor domain in infants with microcephaly related to congenital Zika syndrome.

**Variables**	**Unadjusted effects**	**IC 95%**	**p**	**Adjusted effects**	**IC 95%**	**p**
Mother age (years)	0.239	(-0.166 – 0.644)	0.240			
Maternal symptoms	-5.779	(-12.964 – 1.406)	0.112			
Gestacional age (weeks)	-0.45	(-1.496 – 0.597)	0.391			
Birth weight (g)	0.001	(-0.004 – 0.006)	0.623			
Length at birth (cm)	-0.039	(-0.774 – 0.695)	0.914			
HC at birth (cm)	0.103	(-1.032 – 1.23)	0.856			
zHC at birth	0.930	(-0.745 – 2.606)	0.269	1.44	(-0.225 – 3.12)	0.088
Apgar 1'	0.594	(-1.16 – 2.34)	0.498			
Apgar 5'	-2.70	(-7.92 – 2.51)	0.302			
Female Sex	0.23	(-4.77 – 5.23)	0.927			
Arthrogryposis	-0.167	(-7.56 – 7.23)	0.964			
Low vision	0.34	(-4.76 – 5.44)	0.890			
Neonatal seizure	4.681	(-1.18 – 10.55)	0.115			
Neonatal jaundice	3.24	(-4.69 – 11.19)	0.410			
Neonatal dysphagia	0.515	(-7.44 – 6.41)	0.880			
Ventriculomegaly	-0.602	(-6.87 – 5.67)	0.840			
Colpocephaly	5.186	(-3.51 – 13.88)	0.236			
Corpus callosum dysgene	1.59	(-3.43 – 6.63)	0.520			
Cerebellar hypoplasia	6.88	(-1.70 – 15.47)	0.113			
Evans' Index*	1.883	(-0.1175 – 3.884)	0.064	**2.372**	**(3.01 – 44.4)**	**0.026**

***per 0.1 increase in Evans' Index.**

Table S5. Variables associated with developmental delay in fine motor in infants with microcephaly related to congenital Zika syndrome.

**Variables**	**Unadjusted effects**	**IC 95%**	**p**	**Adjusted effects**	**IC95%**	**p**
Mother age (years)	0.168	(-0.345 – 0.681)	0.513			
Maternal symptoms	-6.183	(-15.20 – 2.835)	0.174			
Gestacional age (weeks)	-0.79	(-2.09 – 0.493)	0.219			
Birth weight (g)	0.001	(-0.006 – 0.007)	0.852			
Length at birth (cm)	-0.151	(-1.064 – 0.762)	0.740			
HC at birth (cm)	-0.187	(-1.59 – 1.22)	0.791			
zHC at birth	0.973	(-1.122 – 3.068)	0.354	1.478	(-0.65 – 3.61)	0.169
Apgar 1'	0.16	(-2.04 – 2.36)	0.883			
Apgar 5'	4.38	(-10.85 – 2.078)	0.178			
Female Sex	2.28	(-3.90 – 8.47)	0.461			
Arthrogryposis	0.965	(-8.24 – 10.17)	0.834			
Low vision	1.611	(-4.72 – 7.94)	0.611			
Neonatal seizure	5.818	(-1.49 – 13.128)	0.116			
Neonatal jaundice	3.78	(-6.11 – 13.68)	0.445			
Neonatal dysphagia	0.026	(-8.66 – 8.61)	0.995			
Ventriculomegaly	-1.045	(-8.8 – 6.76)	0.789			
Colpocephaly	5.804	(-5.063 –16.671)	0.288			
Corpus callosum dysgenesis	3.185	(-3.03 – 9.411)	0.308			
Cerebellar hypoplasia	7.42	(-3.35 – 18.201)	0.172			
Evans' Index*	2.549	(0.022 – 5.077)	0.048	**3.14**	**(5.04 – 57.8)**	**0.021**

***per 0.1 increase in Evans' Index.**
